# α-Bromoacryloyl derivative of distamycin A (PNU 151807): a new non-covalent minor groove DNA binder with antineoplastic activity

**DOI:** 10.1038/sj.bjc.6690453

**Published:** 1999-06

**Authors:** S Marchini, M Cirò, F Gallinari, C Geroni, P Cozzi, M D'Incalci, M Broggini

**Affiliations:** Molecular Pharmacology Unit, Department of Oncology, Istituto di Ricerche Farmacologiche, ‘Mario Negri’ via Eritrea 62, 20157 Milan 20157 Milan, Italy; Pharmacia & Upjohn, Nerviano, Italy

**Keywords:** minor groove binders, cyclin-dependent kinases, DNA binding, anticancer agents

## Abstract

PNU 151807 is a new synthetic α-bromoacryloyl derivative of distamycin A. In the present study we investigated the DNA interaction and the mechanism of action of this compound in parallel with the distamycin alkylating derivative, tallimustine. PNU 151807 possesses a good cytotoxic activity in in vitro growing cancer cells, even superior to that found for tallimustine. By footprinting experiments we found that PNU 151807 and tallimustine interact non-covalently with the same AT-rich DNA regions. However, differently from tallimustine, PNU 151807 failed to produce any DNA alkylation as assessed by *Taq* stop assay and N3 or N7-adenine alkylation assay in different DNA sequences. PNU 151807, like tallimustine, is able to induce an activation of p53, and consequently of p21 and BAX in a human ovarian cancer cell line (A2780) expressing wild-type p53. However, disruption of p53 function by HPV16-E6 does not significantly modify the cytotoxic activity of the compound. Flow cytometric analysis of cells treated with equitoxic concentrations of PNU 151807 and tallimustine showed a similar induction of accumulation of cells in the G2 phase of the cell cycle but with a different time course. When tested against recombinant proteins, only the compound PNU 151807 (and not tallimustine or distamycin A) is able to abolish the in vitro kinase activity of CDK2–cyclin A, CDK2–cyclin E and cdc2–cyclin B complexes. The results obtained showed that PNU 151807 seems to have a mechanism of action completely different from that of its parent compound tallimustine, possibly involving the inhibition of cyclin-dependent kinases activity, and clearly indicate PNU 151807 as a new non-covalent minor groove binder with cytotoxic activity against cancer cells. © 1999 Cancer Research Campaign


*British Journal of Cancer* (1999) **80**(7), 991–997
© 1999 Cancer Research Campaign
Article no. bjoc.1999.0453

### α-Bromoacryloyl derivative of distamycin A (PNU 151807): a new non-covalent minor groove DNA binder with antineoplastic activity

**S Marchini[1], M Cirò[1], F Gallinari[1], C Geroni[2], P Cozzi[2], M D'Incalci[1] and M Broggini[1]**

[1]Molecular Pharmacology Unit, Department of Oncology, Istituto di Ricerche Farmacologiche, 'Mario Negri' via Eritrea 62, 20157 Milan, Italy;
[2]Pharmacia & Upjohn, Nerviano, Italy

**Summary** PNU 151807 is a new synthetic α-bromoacryloyl derivative of distamycin A. In the present study we investigated the DNA interaction and the mechanism of action of this compound in parallel with the distamycin alkylating derivative, tallimustine. PNU 151807 possesses a good cytotoxic activity in in vitro growing cancer cells, even superior to that found for tallimustine. By footprinting experiments we found that PNU 151807 and tallimustine interact non-covalently with the same AT-rich DNA regions. However, differently from tallimustine, PNU 151807 failed to produce any DNA alkylation as assessed by *Taq* stop assay and N3 or N7-adenine alkylation assay in different DNA sequences. PNU 151807, like tallimustine, is able to induce an activation of p53, and consequently of p21 and BAX in a human ovarian cancer cell line (A2780) expressing wild-type p53. However, disruption of p53 function by HPV16-E6 does not significantly modify the cytotoxic activity of the compound. Flow cytometric analysis of cells treated with equitoxic concentrations of PNU 151807 and tallimustine showed a similar induction of accumulation of cells in the G2 phase of the cell cycle but with a different time course. When tested against recombinant proteins, only the compound PNU 151807 (and not tallimustine or distamycin A) is able to abolish the in vitro kinase activity of CDK2–cyclin A, CDK2–cyclin E and cdc2–cyclin B complexes. The results obtained showed that PNU 151807 seems to have a mechanism of action completely different from that of its parent compound tallimustine, possibly involving the inhibition of cyclin-dependent kinases activity, and clearly indicate PNU 151807 as a new non-covalent minor groove binder with cytotoxic activity against cancer cells.

**Keywords:** minor groove binders; cyclin-dependent kinases; DNA binding; anticancer agents

Minor groove alkylating agents are a relatively new class of anti-cancer agents reported to possess high cytotoxic activity in in vitro and in vivo preclinical models (Li et al, 1982, 1992; Hartley et al, 1988; D'Incalci et al, 1997). Their mode of action, although not completely clarified, is likely to involve the formation of adducts, mainly at adenine N3 in the minor groove of DNA (Hurley et al, 1984; Reynolds et al, 1985; Sun et al, 1992; D'Incalci, 1994; Broggini et al, 1995). For all the compounds tested so far, the interaction with DNA and the binding to the N3 of adenines has been reported to be highly sequence-specific (Lee et al, 1993; D'Incalci, 1994), contrasting with the relatively low sequence-specific DNA interaction previously reported for classical major groove alkylating agents (Hartley et al, 1986; Mattes et al, 1986). Tallimustine, a benzoyl mustard derivative of distamycin A, presented a particularly strong sequence-specific DNA interaction, being able to alkylate N3 of adenines only when they are present in the sequence 5′-TTTTGA (Broggini et al, 1991, 1995). These data have been confirmed in intact cells exposed to pharmacologically relevant drug concentrations (Beccaglia et al, 1996). Furthermore, for tallimustine derivatives closely structurally related, the cyto-toxic activity in vitro was found to correlate with their ability to alkylate N3 adenine in the above-mentioned sequence (Marchini et al, 1998).

*Received 17 July 1998*
*Revised 4 January 1999*
*Accepted 6 January 1999*

*Correspondence to:* M Broggini

The absence of significant anti-tumour activity for the non-alkylating minor groove binder distamycin, from which tallimus-tine was derived, further supports the evidence that N3 alkylation is a pre-requisite for the cytotoxic activity of these compounds. This was also shown for new tallimustine derivatives which were not able to alkylate DNA and also did not exert cytotoxic activity in vitro (Marchini et al, 1998).

Tallimustine was effective against several rodent and human tumours, but in humans it showed a very high bone marrow toxicity (Sessa et al, 1994; D'Incalci et al, 1997; Ghielmini et al, 1997). A series of other distamycin derivatives was synthesized and tested for anti-tumour activity and bone marrow toxicity: one possessing a favourable therapeutic index (i.e. high anti-tumour activity with relatively low bone marrow toxicity) was the α-bromoacryloyl derivative of distamycin A (PNU 151807) (D'Alessio et al, 1994; Ghielmini et al, 1997). We report here the characterization of the interaction of this new compound with DNA and studies on its mechanism of action.

### MATERIALS AND METHODS

### Cells

The human ovarian carcinoma cell line A2780 and its subline A2780/E6 (Vikhanskaya et al, 1998), derived from parental cells by transfection with the DNA encoding for the E6 protein of the human papillomavirus 16 (HPV16) under the control of the cytomegalovirus (CMV) promoter, the B16F10 murine melanoma, the murine lymphocytic leukaemia L1210 and the human T-cell

**Table 1** Cytotoxicity of PNU 151807 and tallimustine ($IC_{50}$, ng $ml^{-1}$)

| Cell line | PNU 151807 | Tallimustine | R |
|---|---|---|---|
| L1210 | 9.64 ± 1.37 | 84.8 ± 12 | 8.79 |
| B16F10 | 29.64 ± 2.4 | 1096 ± 286 | 36.97 |
| Jurkat | 22.85 ± 2.3 | 13.8 ± 2.4 | 0.6 |
| CEM | 19.8 ± 3.4 | 10.2 ± 1.8 | 0.5 |
| HT29 | 658.5 ± 77 | 4177 ± 928 | 6.3 |
| LoVo | 106.4 ± 21 | 826 ± 155 | 7.79 |
| A2780 | 12.54 ± 4.3 | 378 ± 16 | 30 |
| DU145 | 69.19 ± 1.8 | 55.6 ± 27 | 0.8 |

R = Ratio between $IC_{50}$ of tallimustine and PNU 151807.

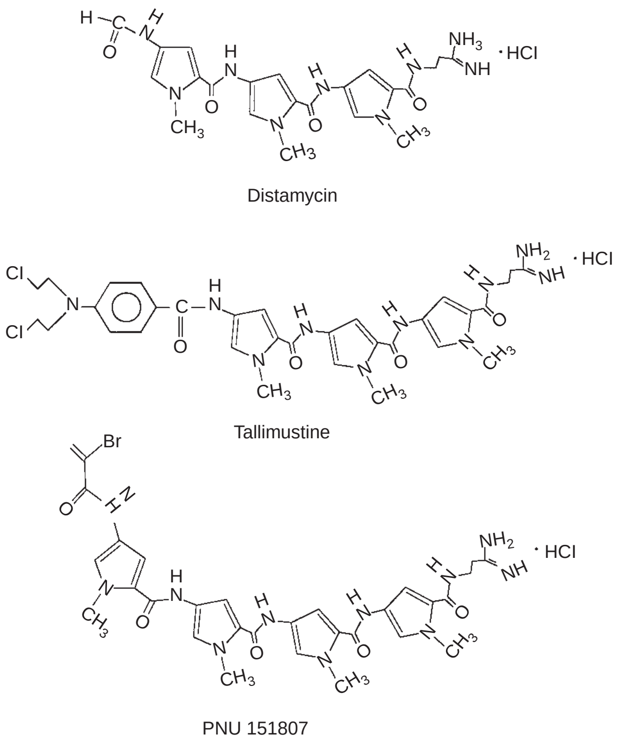

**Figure 1** Chemical structure of distamycin A, tallimustine and PNU 151807

leukaemia Jurkat were maintained in RPMI-1640. The human lymphoblastic leukaemia cell line CEM was maintained in EMEM (Bio Whittaker), the human colon adenocarcinoma LoVo and HT29 in Ham's F12 medium (Bio Whittaker) and the human prostatic carcinoma DU145 in DMEM. All media were supplemented with 10% fetal calf serum (FCS).

### In vitro drug sensitivity

In vitro drug sensitivity was determined for LoVo, HT29, DU 145, B16F10 and A2780 cells by using the sulphorhodamine B assay. Exponentially growing cells were seeded 24 h before treatment, exposed to drugs for 1 h and then incubated in drug-free medium for 72 h. In vitro drug sensitivity against CEM, Jurkat and L1210 cells was evaluated by counting surviving cells. Exponentially growing cells were seeded and exposed to various concentrations of drugs immediately after seeding. The incubation mixture was kept at 37°C for 1 h, after which the cells were washed and incubated for 72 h (48 h for L1210) in drug-free medium. The antiproliferative activity of the drugs was calculated from dose–response curves and expressed as $IC_{50}$ (dose causing 50% inhibition of cell growth in treated cultures relative to untreated controls).

### Cell cycle analysis

A2780 cells were treated with equitoxic concentrations of tallimustine and PNU 151807 for 1 h. Monoparametric cell cycle analysis on ethanol-fixed cells using propidium iodide was carried out on control and treated cells at different times after drug washout. Each cytometric DNA analysis was performed on the FACSSort (Becton Dickinson) on 1–2 × $10^4$ cells and the percentage of cells in the different cell cycle phases was evaluated as previously described (Broggini et al, 1991).

### Sequence specificity of adenine $N^3$ and $N^7$ alkylation

The method has been previously described in detail (Reynolds et al, 1985; Marchini et al, 1995). Briefly, *Eco*RI (NEB) digested SV 40 viral DNA (Gibco, BRL) was $\gamma$-$^{32}$P-labelled at its 5′ ends with T4 polynucleotide kinase (NEB). A second cut with *Bam*HI (NEB) was performed to produce a 4492 and a 751 bp fragments labelled at only one end. Alkylations were performed in sodium citrate–sodium chloride (SSC) 0.1 × buffer at room temperature for 5 h at doses selected to give approximately one alkylation per DNA molecule. After precipitation and washing, the DNA was heated at 90°C for 30 min in 0.1 × SSC buffer for N3 alkylation assay, while for N7 alkylation assay DNA was heated for 15 min at 90°C in 1 M piperidine and then washed twice with water. DNA fragments were separated on 0.4 mm, 8% polyacrylamide gels containing 7 M urea and a Tris–boric acid EDTA buffer system. Gels were run at 55 W (approximately 3000 V) for 3 h, dried and autoradiographed. DNA sequencing was performed according to the Maxam and Gilbert assay.

### MPE footprinting

The 4492- and 751-bp labelled fragments previously described were incubated with different concentrations of drugs for 1 h at room temperature and then treated with a solution of MPE-$(NH_4)_2$-$Fe(SO_4)_2.6H_2O$ (synthetized by Pharmacia & Upjohn according to published method) (Hertzberg et al, 1984) for 30 min at room temperature. After precipitation, DNA was resuspended in loading buffer and electrophoresed on 8% polyacrylamide–7 M urea gels and autoradiographed.

### *Taq* polymerase stop assay

The procedure employed was an application of a previously described method (Ponti et al, 1991). Prior to drug-DNA incubation, plasmid pBSSK-TOPO II was linearized with *Pst*I enzyme (NEB) to provide a stop for the *Taq* polymerase downstream from the primer. After drug treatment, the DNA was precipitated and washed as described (Ponti et al, 1991). The primers were 5′-end-labelled prior to amplification using $T_4$ polynucleotide kinase (NEB) and $\gamma$-$^{32}$P-ATP (5000 Ci $mmol^{-1}$, Amersham). The synthetic primer sequence and the polymerase chain reaction (PCR) linear DNA amplification were performed as described (Ponti et al, 1991; Marchini et al, 1998). Samples were then purified and

 *© 1999 Cancer Research Campaign*

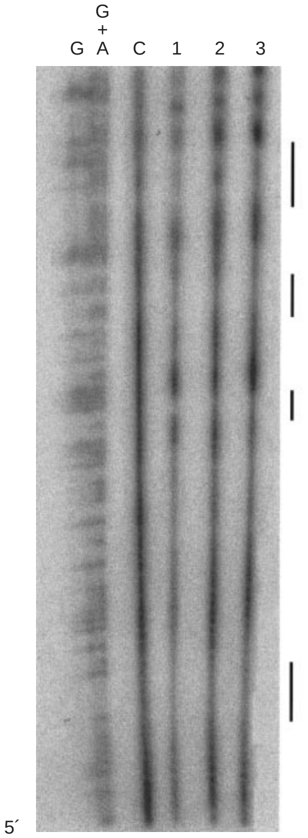

**Figure 2** MPE footprinting analysis in the 751-bp *Eco*RI–*Bam*H1 fragment of SV40. G and G+A are Maxam and Gilbert sequencing reaction for guanines and guanines plus adenines, respectively. Lane 1, distamycin A (250 μM); lane 2, tallimustine (250 μM); lane 3, PNU 151807 (250 μM). Protected regions are evidentiated by a mark on the right side. C: control, untreated DNA

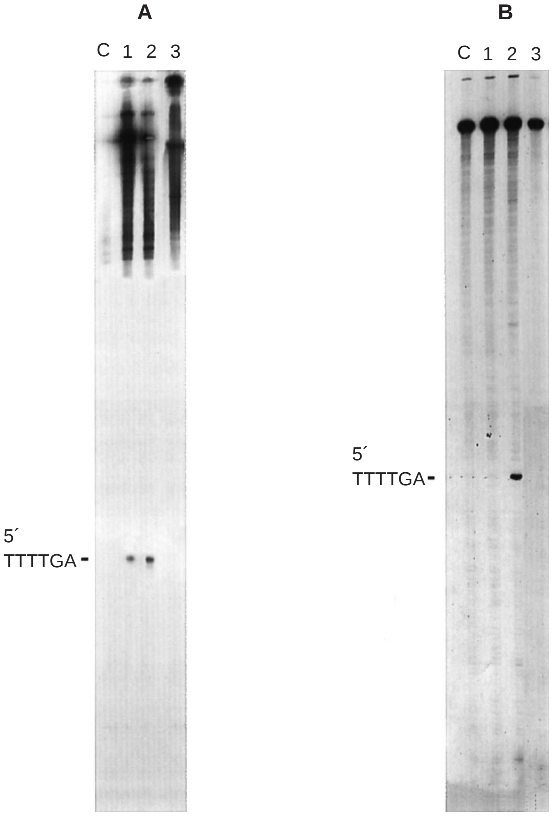

**Figure 3** DNA alkylation assay. (**A**) Sites of N3 adenine alkylation produced by drugs in *Eco*RI-digested SV40 DNA, $^{32}$P-labelled at the 5′ end and further cleaved with *Bam*HI. Lane 1, tallimustine (250 μM); lane 2, tallimustine (500 μM); lane 3, PNU151807 (500 μM). (**B**) *Taq* polymerase stop assay on pBSSK-TOPO II DNA incubated with different drugs. Lane 1, distamycin (10 μM); lane 2, tallimustine (10 μM); lane 3, PNU 151807 (10 μM)

loaded on 8% polyacrylamide denaturing gel. After the run, the gel was dried and autoradiographed.

### Western blot analysis

Total cell extracts were prepared from untreated or treated cells at different times after drug exposure, according to standard procedures (Sambrook et al, 1989). 20 μg of proteins for each sample were electrophoresed through 12% polyacrylamide-sodium dodecyl sulphate (SDS) gels and electroblotted onto nitrocellulose membrane (Schleicher & Schull, Germany) in transfer buffer (50 mM Tris, 100 mM glycine, 0.01% SDS, 20% methanol) for 2 h at 50 V. Filters were stained with Ponceau red, hybridized with monoclonal antibody against p53 (clone DO-1), and detected with the enhanced chemiluminiscence system (ECL). The experiments were repeated in all the cell lines at least twice.

### Northern blot analysis

Total RNA was extracted from untreated or treated cells with the guanidine/caesium chloride gradient method (Sambrook et al, 1989). After fractionation through 1% agarose-formaldehyde gels, RNA was blotted on nylon membranes (GeneScreen plus, Dupont) and hydridized with cDNAs encoding bax (kindly supplied by Dr Korsmeyer, St Louis, MO, USA) and WAF1. Each cDNA was $^{32}$P-labelled using a Rediprime kit (Amersham, UK). Hybridizations were performed in 50% formamide, 10% dextran suphate, 1% SDS, 1 M sodium chloride at 42°C for 16 h, followed by two washes at room temperature for 10 min with 2 × SSC (150 mM sodium chloride, 15 mM sodium citrate) and one wash for 30 min at 65°C in 2 × SSC–1% SDS. WAF1 cDNA was obtained by PCR as previously described (Vikhanskaya et al, 1994). Each filter was hybridized with α-actin cDNA to normalize for RNA loading.

### Cyclin-dependent kinase (cdk) activity

Inhibition of kinase activity was measured as previously reported (Bonfanti et al, 1997) by incubating 100 ng of total extracts containing insect cell-expressed cyclin E–cdk2, cyclin A–cdk2 or cyclin B–cdc2 in the presence of different drug concentrations at 4°C for 30 min. Kinase reactions were followed at 30°C for 20 min in a total volume of 25 μl of kinase buffer (50 mM Tris–HCl pH 7.4, 150 mM sodium chloride, 0.5% Triton X-100, 10 mM magnesium chloride, 1 mM dithiothreitol) containing 2 μg of histone H1, 1 μM ATP and 5 μCi [γ-$^{32}$P]ATP (5000 Ci mmol$^{-1}$; Amersham); 25 μl of 2 × SDS-loading buffer were added and the samples were boiled and loaded on 12% SDS polyacrylamide gels. Histone H1 was loaded as a marker of molecular weight and separately stained with Coomassie blue.

© 1999 Cancer Research Campaign 

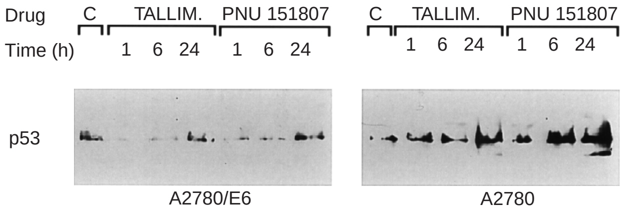

**Figure 4** Western blotting analysis of p53 expression in A2780 and A2780/E6 cells after 1, 6 and 24 h of treatment with the respective $IC_{50}$ of tallimustine and PNU 151807. C: controls, untreated cells

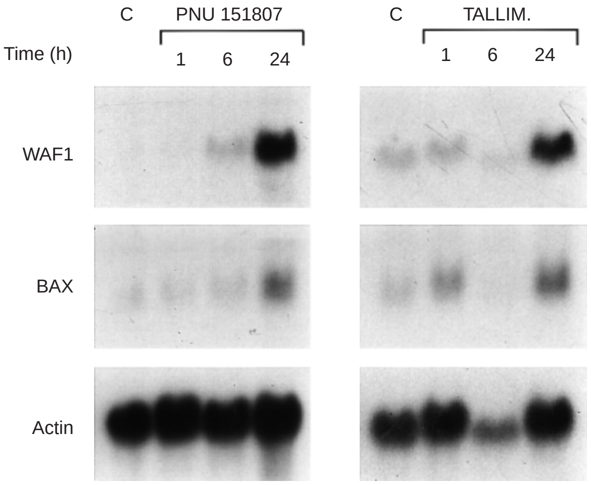

**Figure 5** mRNA expression of WAF1 and BAX in A2780 cells after 1, 6 and 24 h of treatment with tallimustine and PNU 151807 at the respective $IC_{50}$s

### RESULTS

The cytotoxic activity of PNU 151807 in comparison with tallimustine (see structure in Figure 1), evaluated in different cell lines, is reported in Table 1. With the exception of CEM and Jurkat cell lines (in which tallimustine seemed to be twice as potent as PNU 151807) and DU145 (in which the two compounds had roughly the same cytotoxicity), in all the other cancer cell lines tested PNU 151807 showed higher cytotoxicity than the parental alkylating agent tallimustine. Non-covalent DNA interactions of PNU 151807 and tallimustine were initially evaluated by footprinting analysis. Figure 2 reports a representative experiment conducted in parallel with distamycin A and shows that both the compounds tested protect quite the same AT-rich region as distamycin A. Analysis of different DNA fragments and sequences did not reveal any significant difference in the non-covalent DNA interaction among the different compounds. Differences in band intensity may be due to differences in gel loading.

PNU 151807 was then tested for its ability to covalently interact with DNA. By testing different DNA sequences with the N3-adenine alkylation assay, we failed to detect any alkylation. Figure 3A reports an example of these gels in which in the same treatment conditions aklylation by tallimustine was detectable in the target sequence 5′-TTTTGA (increasing the drug-DNA incubation times and the heating treatment to detect breaks at adenine N3 failed to reveal any alkylation, data not shown). The lack of covalent PNU 151807–DNA interaction was further confirmed by *Taq* stop assay which allows the detection also of adducts different from those produced at N3 adenine (as for example those produced at N2-guanine). As we can see from Figure 3B, PNU 151807 is unable to produce any kind of alkylation in the examined sequences. DNA incubation with PNU 151807 was even prolonged to 24 h and tested in different DNA sequences, but again we failed to detect any kind of alkylation (data not shown). Trying to elucidate the mechanism of action of PNU 151807, the human ovarian cancer cell line A2780 and a subline derived from it in which wild-type p53 has been disrupted (A2780/E6) have been selected and tested for the growth inhibition produced by the two compounds. The data obtained showed that the two compounds were only slightly more cytotoxic in cells not expressing p53 being the ratio between the $IC_{50}$ in A2780 and A2780/E6 2.7 and 2.1, respectively, for tallimustine and PNU 151807.

We then evaluated whether treatment of the cells with concentrations close to the $IC_{50}$ was able to activate p53 and subsequently p21 and bax genes. By Western blotting analysis, we could detect a clear induction of p53 after treatment with both compounds in A2780 cells (Figure 4). As expected in A2780/E6 both compounds did not induce the expression of p53. The increase in the p53 protein levels, observed in A2780 cells, was associated with an increase in the mRNA levels of two genes transcriptionally regulated by p53, i.e. WAF1 and, to a lesser extent BAX (Figure 5).

The cell cycle perturbation induced by PNU 151807 and tallimustine was evaluated at different times of incubation in drug-free medium after 1 h treatment with equitoxic concentrations in A2780 cells. As reported in Figure 6 both compounds produced an accumulation of cells in G2, evident after 24 h incubation in drug-free medium for PNU 151807 and later (48 h after incubation) for tallimustine.

To further analyse PNU 151807 effects on cell cycle proteins, we tested whether both PNU 151807 and tallimustine were able to interfere with the kinase activity of cdk. We incubated different concentrations of the compounds with baculovirus produced recombinant CDK2–cyclin A, CDK2–cyclin E and cdc2–cyclin B, and then evaluated the kinase activity measured as ability to phosphorylate in vitro histone H1. As shown in Figure 7, PNU 151807 inhibited CDK2 kinase activity when complexed with cyclin A or cyclin E. At the same concentrations tested, neither tallimustine nor distamycin A caused inhibition of the kinase activity, with only marginal effect observable at the highest concentrations. The same was observable for cdc2 whose activity was inhibited at significant extent only by PNU 151807. The experiments were repeated at least three times for each kinase with essentially the same results.

### DISCUSSION

Minor groove alkylating binders are a new class of anticancer agents reported to possess high anti-tumour activity in both in vitro and in vivo experimental models (D’Incalci, 1994). In particular, a number of distamycin A derivatives have been tested for their cytotoxic activity and compared with tallimustine, the first alkylating distamycin derivative entered into phase I clinical trials (Sessa et al, 1994). The activity of this class of compounds has been related to their ability to alkylate DNA, in a very high sequence-specific manner with the N3 of adenine in the minor groove as main target (Coley et al, 1990; Broggini et al, 1991, 1995). In general, loss of alkylating ability has been associated with a dramatic loss of cytotoxic activity in vitro (Marchini et al,

*© 1999 Cancer Research Campaign*

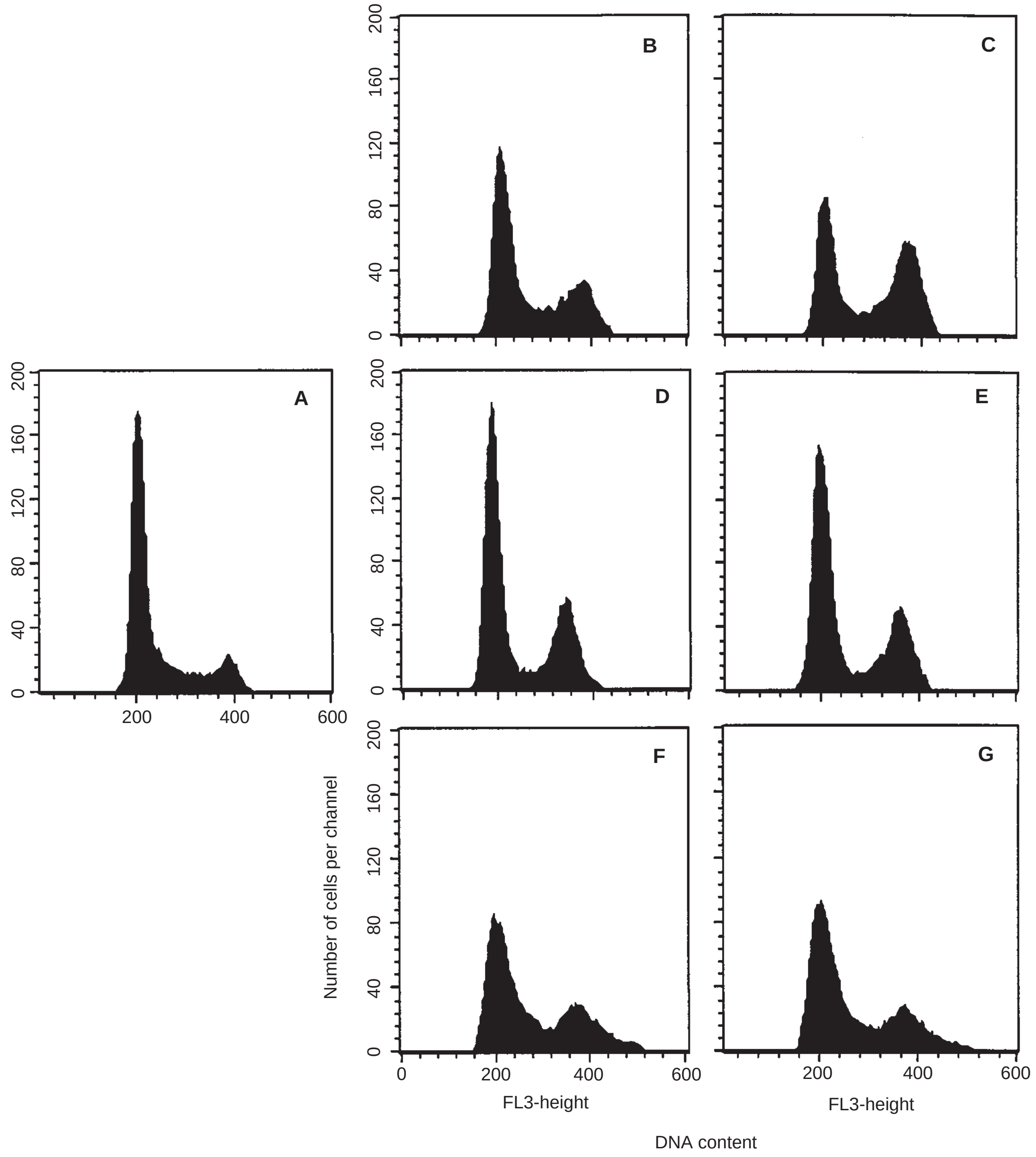

**Figure 6** Cell cycle perturbations induced by PNU 151807 (panels **C**, **E**, **G**) and tallimustine (panels **B**, **D**, **F**) in A2780 cells treated with the respective $IC_{50}$s for 1 h. Analysis were performed after 24 (panels **B**, **C**), 48 (panels **D**, **E**) and 72 (panels **F**, **G**) h incubation in drug-free medium. Controls, untreated cells are in panel (**A**)

1998), suggesting that the mechanism of action is related to the covalent DNA interaction occurring between the alkylating moiety of the compounds and the N3-adenine of DNA.

The α-bromoacryloyl derivative of distamycin A PNU 151807, is clearly an exception to these observations, as it has equal or even superior cytotoxic activity when compared to tallimustine, but does not appear to covalently interact with DNA as indicated by the DNA alkylation experiments reported here.

These data fit with the chemical available data which indicate a poor reactivity of the bromoacryloyl group when linked to the distamycin moiety. In fact, the molecule was proved to be stable at room temperature for 3 days at pH 12. Moreover, it was stable when heated at 60°C for 48 h in acetone:water at pH 10, conditions leading to hydrolysis of nitrogen mustards, even of those with low reactivity such as tallimustine (P Cozzi et al, manuscript in preparation). The lack of covalent interaction was not due to inability of the modified distamycin backbone to interact with DNA, since the footprinting experiments revealed that PNU 151807 protected the same DNA regions protected by tallimustine and distamycin A. Since distamycin A is devoid of any anti-tumour activity, unless utilizing very high concentrations (Geroni et al, 1993), the bromoacryloyl part of the molecule must have a biological relevance for cytotoxicity.

Both tallimustine and PNU 151807 are able to increase the levels of p53 in A2780 cells, and this increase was associated to an increased transcriptional activation as judged by the raise in the mRNA levels of WAF1 gene. Nevertheless, the presence of p53 does not play a significant role in determining the cytotoxicity of these two compounds in A2780 cells. Utilizing a clone obtained from A2780 cells and previously reported to have disruption of p53 function (Vikhanskaya et al, 1998), we only observed a slight

*© 1999 Cancer Research Campaign* 

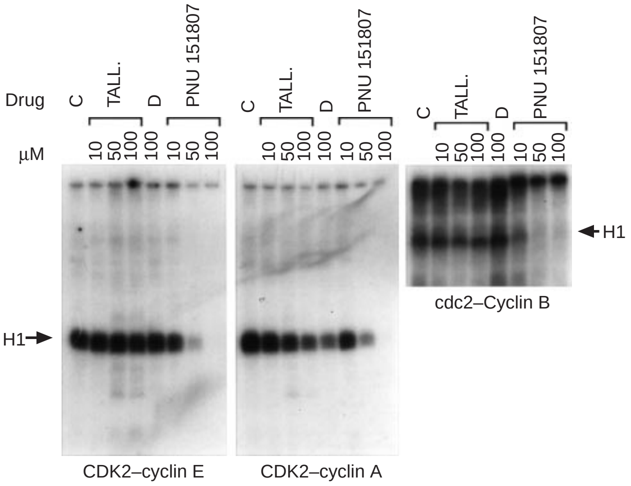

**Figure 7** Effect of PNU 151807, tallimustine (tall.) and distamycin A (D) on cyclin-dependent kinase activity. CDK2–cyclin A, CDK2–cyclin E and cdc2–cyclin B complexes were incubated with different concentrations of the compounds as indicated before the addition of histone H1, substrate of the three kinases. C is the activity of the cyclin–kinase complex in the absence of drug increase in the activity of both compounds, while in the same system we have reported that the widely used anticancer agent taxol showed a markedly increased (roughly 50 times) activity in A2780/E6 cells compared with the parental cell line (Vikhanskaya et al, 1998). Nevertheless, the increase in p53 levels induced by PNU 151807 does not necessarily imply that DNA damage is occurring as other compounds not directly interacting with DNA are also able to do it (Tishler et al, 1995).

It has been reported that some compounds with anti-tumour activity were able to interfere with the phosphorylating activity of cdk (Drees et al, 1997), and the known cdks are under investigation as possible targets for the synthesis of new anticancer agents (Bonfanti et al, 1997).

PNU 151807 is able to inhibit the activity of the complexes CDK2–cyclin A, CDK2–cyclin E and CDC2–cyclin B when tested against recombinant proteins in vitro. These findings open the possibility that the drug action could be through the inhibition of these important cell cycle controlling proteins. This effect appears to be independent from the DNA binding activity since tallimustine or distamycin A only modestly, and at the highest concentrations tested, interfere with this activity.

In conclusion, PNU 151807 is a new promising anticancer agent with a mechanism of action different from the previously tested alkylating minor groove binders (D'Incalci, 1994). Considering that some of the latter compounds were associated with unexpected clinical haematologycal toxicity, and that from studies on haematologycal precursors maintained in vitro it appears that PNU 151807 had a much lower toxicity and a higher therapeutic index than tallimustine (Ghielmini et al, 1997), PNU151807 or analogues of it could represent an interesting clinical alternative to alkylating minor groove binders.

### ACKNOWLEDGEMENTS

We would like to thank Dr Piwnica-Worms for baculovirus expressing cyclin B and cdc2 and Dr Morgan for baculovirus expressing cyclin A, cyclin E and CDK2. The generous contribution of the Italian Association for Cancer Research is gratefully acknowledged. SM is recipient of a fellowship from Italian Foundation for Cancer Research (FIRC).

### REFERENCES

Beccaglia P, Grimaldi KA, Hartley JA, Marchini S, Broggini M and D'Incalci M (1996) DNA adduct formation of the sequence selective cytotoxic agent tallimustine resolved at the nucleotide level in a single copy gene in mammalian cells. *Br J Cancer* **73**: 12

Bonfanti M, Taverna S, D'Incalci M and Broggini M (1997) p21 WAF-derived peptides linked to an internalization peptide inhibit human cancer cell growth. *Cancer Res* **57**: 1442–1446

Broggini M, Erba E, Ponti M, Ballinari D, Geroni C, Spreafico F and D'Incalci M (1991) Selective DNA interaction of the novel distamycin derivative FCE 24517. *Cancer Res* **51**: 199–204

Broggini M, Coley HM, Mongelli N, Pesenti E, Wyatt MD, Hartley JA and D'Incalci M (1995) DNA sequence-specific adenine alkylation by the novel antitumor drug tallumustine (FCE 24517), a benzoyl nitrogen mustard derivative of distamycin. *Nucleic Acids Res* **23**: 81–87

Coley HM, Broggini M and D'Incalci M (1990) Studies of the novel distamycin compound FCE 24517 with respect to DNA interaction and sensitivity to alkylating agents. *Br J Cancer* **62**: 506–500

D'Alessio R, Geroni C, Biasoli G, Pesenti E, Grandi M and Mongelli N (1994) Structure activity relationship of novel distamycin A derivatives: synthesis and antitumor activity. *Bioorganic Med Chem Lett* **4**: 1467–1472

D'Incalci M (1994) DNA-minor groove alkylators, a new class of anticancer agents. *Ann Oncol* **5**: 877–878

D'Incalci M and Sessa C (1997) DNA minor groove binding ligands: a new class of anticancer agents. *Exp Opin Invest Drugs* **6**: 875–884

Drees M, Dengler WA, Roth T, Labonte H, Mayo J, Malspeis L, Grever M, Sausville EA and Fiebig HH (1997) Flavopiridol (L86-8275): selective antitumor activity in vitro and activity in vivo for prostate carcinoma cells. *Clin Cancer Res* **3**: 273–279

Geroni C, Pesenti E, Tagliabue G, Ballinari D, Mongelli N, Broggini M, Erba E, D'Incalci M, Spreafico F and Grandi M (1993) Establishment of L1210 leukemia cells resistant to the distamycin-A derivative (FCE 24517): characterization and cross-resistance studies. *Int J Cancer* **53**: 308–314

Ghielmini M, Bosshard G, Capolongo L, Geroni C, Pesenti E, Torri V, D'Incalci M, Cavalli F and Sessa C (1997) Estimation of the haematological toxicity of minor groove alkylators using tests on human cord blood cells. *Br J Cancer* **75**: 878–883

Hartley JA, Gibson NW, Kohn KW and Mattes WB (1986) DNA sequence selectivity of guanine-N7 alkylation by three antitumor chloroethylating agents. *Cancer Res* **46**: 1943–1947

Hartley JA, Lown JW, Mattes WB and Kohn KW (1988) DNA sequence specificity of antitumor agents. Oncogenes as possible targets for cancer therapy. *Acta Oncol* **27**: 503–510

Hertzberg RP and Dervan PB (1984) Cleavage of DNA with Methidiumpropyl-EDTA-Iron(II): reaction conditions and product analyses. *Biochemistry* **23**: 3934–3945

Hurley LH, Reynolds VL, Swenson DH, Petzold GL and Scahill TA (1984) Reaction of the antitumoral antibiotic CC-1065 with DNA: structure of DNA adduct with sequence specificity. *Science* **226**: 843–844

Lee M, Rhodens L, Wyatt MD, Forrow S and Hartley JA (1993) Design, synthesis, and biological evaluation of DNA sequence and minor groove selective alkylating agents. *Anticancer Drug Des* **8**: 173–192

Li LH, Swenson D, Schpock S, Kuentzel S, Dayton B and Kreiger W (1982) CC-1065 (NSC-298223) a novel antitumour agent that interacts strongly with double-stranded DNA. *Cancer Res* **42**: 999–1004

Li LH, Dekoning TF and Kelly RC (1992) Cytotoxicity and antitumor activity of carzelesin, a prodrug cyclopropylpyrroloindole analogue. *Cancer Res* **52**: 4904–4913

Marchini S, Gonzales Paz O, Ripamonti M, Geroni C, Bargiotti A, Caruso M, Todeschi S, D'Incalci M and Broggini M (1995) Sequence-specific DNA interactions by novel alkylating anthracycline derivatives. *Anticancer Drug Des* **10**: 641–653

Marchini S, Cozzi P, Beria I, Geroni C, Capolongo L, D'Incalci M and Broggini M (1998) Sequence specific DNA alkylation of novel tallimustine derivatives. *Anticancer Drug Des* **13**: (in press)

Mattes WB, Hartley JA and Kohn KW (1986) DNA sequence selectivity of guanine-N7 alkylation by nitrogen mustards. *Nucleic Acids Res* **14**: 2971–2987

*© 1999 Cancer Research Campaign*

Ponti M, Forrow SM, Souhami RL, D'Incalci M and Hartley JH (1991) Measurement of the sequence specificity of covalent DNA modification by antineoplastic agents using Taq DNA polymerase. *Nucleic Acids Res* **19**: 2929–2933

Reynolds VL, Molineux IJ, Kaplan DJ, Swenson DH and Hurley LH (1985) Reaction of the antitumor antibiotic CC-1065 with DNA. Location of the site of thermally induced strand breakage and analysis of DNA sequence specificity. *Biochemistry* **24**: 6228–6237

Sambrook J, Fritsh E and Maniatis T (1989) *Molecolar Cloning: A Laboratory Manual*, Cold Spring Harbor, NY: Cold Spring Harbor Laboratory Press

Sessa C, Pagani O, Zurlo MG, Jong DE, Hoffmann C, Lassus M, Marrari P, Strolin, Benedetti M and Cavalli F (1994) Phase I study of the novel distamycin derivative tallimustine (FCE 24517). *Ann Oncol* **5**: 901–907

Sun D and Hurley LH (1992) Effect of the (+)-CC-1065-(N3-adenine) DNA adduct on in vitro DNA synthesis mediated by *Escherichia coli* DNA polymerase. *Biochemistry* **31**: 2822–2829

Tishler RB, Lamppu DM, Park S and Price BD (1995) Microtubule-active drugs taxol, vinblastine, and nocodazole increase the levels of transcriptionally active p53. *Cancer Res* **55**: 6021–6025

Vikhanskaya F, Erba E, D'Incalci M and Broggini M (1994) Introduction of wild-type p53 in human ovarian cancer cell line not expressing endogenous p53. *Nucleic Acids Res* **22**: 1012–1017

Vikhanskaya F, Vignati S, Beccaglia P, Ottoboni C, Russo P, D'Incalci M and Broggini M (1998) Inactivation of p53 in a human ovarian cancer cell line increases the sensitivity to paclitaxel by inducing G2/M arrest and apoptosis. *Exp Cell Res* **241**: 96–101

*© 1999 Cancer Research Campaign*
